# Exploring the association between phase angle of bioimpedance at 50 kHz and cardiovascular risk

**DOI:** 10.1186/s12872-024-04211-4

**Published:** 2024-10-29

**Authors:** Evandro Lucas de Borba, Cristina Wichbold, Jamile Ceolin, Marcelo Rodrigues Gonçalves, Wilson Cañon-Montañez, Alexandre Vontobel Padoin, Rita Mattiello

**Affiliations:** 1 Medical School, Pontifícia Universidade Católica do Rio Grande do Sul, PUCRS, Porto Alegre, Brazil; 2 Medical School, Universidade do Vale do Rio dos Sinos, UNISINOS, São Leopoldo, Brazil; 3 Faculty of Nursing, Centro Universitário Metodista - IPA, Porto Alegre, Brazil; 4 Medical School, Universidade Federal do Rio Grande do Sul, UFRGS, R. Ramiro Barcelos, 2400 - Santa Cecília, Porto Alegre, RS, 90035-002 Brazil; 5 Faculty of Nursing, Universidad de Antioquia, Medellín, Colombia

**Keywords:** Bioelectrical Impedance Analysis, Cardiovascular Disease, Phase Angle, Cardiovascular Risk Calculator

## Abstract

**Background:**

Cardiovascular diseases are characterized by chronic inflammation, leading to increased inflammatory markers that can cause cell damage and death. Phase angle has emerged as a marker of cellular health. It is considered a prognostic factor in various acute and chronic conditions. However, few studies have examined its association with cardiovascular disease risk measures. This study aims to investigate the relationship between phase angle, the general Framingham risk score, and the HEARTS cardiovascular risk score.

**Methods:**

This cross-sectional study included a convenience sample of adult patients of 2 primary health care services. Phase angle was measured using multifrequency bioimpedance analysis at 50 kHz. The risk of cardiovascular events was calculated using the Framingham and HEARTS risk scores. Statistical analysis included generalized linear regression models, unadjusted and adjusted according to sex and age, to determine the association between scores, risk factors, and phase angle.

**Results:**

The study included 164 individuals with a mean age 52.2 (SD 17.9). According to the HEARTS score, low-risk patients had higher phase angle values than those with high or very high risk [ß = -0.57 (95% CI -0.95; -0.19), *P* = 0.003]. Framingham scores showed a trend toward significance for higher mean phase angle values in low-risk than high-risk patients [ß = -0.43 (95% CI -0.88 to 0.02), *P* = 0.06].

**Conclusion:**

Phase angle values were lower in high and very high-risk patients than in low-risk patients, which shows that phase angle is a promising risk predictor for patients with cardiovascular diseases.

## Introduction

Cardiovascular disease (CVD) remains the leading cause of morbidity and mortality [1]. In 2019, CVD caused approximately 17.9 million deaths, i.e., 32% of all deaths worldwide [2].

Since it is a prominent factor contributing to global mortality, timely identification is paramount. Current CVD guidelines recommend using risk prediction tools to identify those at the highest risk of CVD. Risk assessment has thus become critical to preventing CVD by promoting healthier lifestyles, pharmacological and other healthcare interventions, and reducing the prevalence of risk factors. In this context, several multivariate CVD risk models have been developed for global risk estimation across populations [3, 4].

The available risk assessment tools include the Framingham risk score, widely used to estimate the risk of a first cardiovascular event within ten years, and the HEARTS cardiovascular risk calculator, calibrated for use in Latin America and the Caribbean [5, 6]. However, a limiting factor in risk prediction scores is the number of measurements they include [7]. As advancements in cardiovascular risk estimation tools progress, there is an urgent need to enhance these prediction models to ensure their validity across diverse populations. This includes accommodating a broad spectrum of ages and incorporating low-cost, non-invasive, improving accessibility and applicability in various clinical settings.

Recently, a systematic review and meta-analysis indicated that the bioimpedance phase angle (PhA) has emerged as a significant marker of cellular health. It is recognized as a valuable clinical prognostic assessment tool for various acute and chronic diseases [8–14], including those patients with CVD [15].

Healthy cells possess adequate nutrients and energy resources to produce cell membranes with the proper structure and function. This enables them to maintain a charge difference across the membrane and generate an appropriate membrane potential with significant electric capacitance, resulting in a large phase shift of the applied alternating current, referred to as the PhA [16]. Emerging data suggest that dysregulation of inflammatory, oxidative stress, and immune pathways may be a primary mechanism in many CVDs, leading to cell damage and death [17]. Additionally, higher PhA values, which correlate with better outcomes in CVD, are directly associated with increased lean mass and muscle mass [18].

Although PhA has been associated with several diseases, its role as a risk predictor of CVD is still little understood. Nevertheless, some studies have already evaluated a possible association between PhA and general Framingham risk scores. A study by Saad et al. [19] of Brazilian primary care patients aged > 60 years found an independent association between overall cardiovascular risk and age. Portugal et al. [20] evaluated non-teaching civil servants (aged 30 to 74 years) at a Brazilian university, finding higher mean PhA values in low-risk than high-risk individuals. However, few studies have evaluated PhA concerning the HEARTS Risk Score, the first risk calculator calibrated and validated for use in Brazilians, and the independent variables included in the Framingham risk scores.

To better understand PhA as a predictor of CVD, this study aimed to assess the association between PhA and Framingham and HEARTS risk scores and the variables involved in these risk scores.

## Methods

This cross-sectional study followed STROBE guidelines for reporting observational studies [21]. In a convenience sample, we included patients aged ≥ 20 (20 to 93) from 2 primary healthcare services in the greater Porto Alegre region in Rio Grande do Sul, Brazil. We excluded patients with disabling psychiatric or neurological diseases, end-stage liver and or kidney disease, corticosteroid or immunosuppressant users, pregnant women, patients with pacemakers or cardioverter-defibrillators, and amputees or individuals with metallic prostheses/orthotics.

### Outcome variable

Bioimpedance Multifrequency InBodyS10 (Ottoboni, Rio de Janeiro, RJ, Brazil) was utilized to evaluate PhA. The InBodyS10 showed excellent agreements with DEXA (intraclass correlation coefficients > 0.90 for body composition measurements for both men and women) [22]. The applied frequency was 50 kHz. The hand electrodes were attached to each thumb and middle finger, while the foot electrodes were positioned between the ankle bone and the heel, covering as much area as possible. The bioimpedance was performed with the participants on a non-conductive surface in the standing position, with their legs apart and arms held away (15º) from their body and wearing the least amount of clothing possible and no metal jewelry. All the participants completed three evaluations, and the average of the three values was considered as their result. Measurements were performed by one of the four experienced researchers according to the manufacturer’s instructions and prior evidence for applying the bioimpedance [23, 24]. The measurements were carried out in the clinical environment.

### Exposure variables

Cardiovascular risk was assessed using the general Framingham risk score [5], with points being scored for the following variables: age, high-density lipoprotein cholesterol, total cholesterol, systolic blood pressure, smoking, and diabetes [5]. Risk $\:\le\:$ 5%, > 5%, and $\:<$ 20%, $\:\ge\:$ 20% were classified as low, intermediate, and high, respectively [5, 25]. The same variables were used to calculate the HEARTS CVD risk score, except that weight and height could be substituted for lipid profile in patients without one [6]. Risk $\:\le\:$ 5%, > 5% $\:<$ 10%, $\:\ge\:$ 10% < 20%, and $\:\ge\:$ 20% was classified as low, intermediate, high, and very high. In this classification system, kidney failure and diabetes automatically put an individual at high risk [6]. Patients with a prior history of cardiovascular events were considered at high risk in the Framingham system and at very high risk in the HEARTS system [6, 26].

### Covariates

Sociodemographic and clinical variables were collected using a standardized questionnaire with the following information: age (categorized as $\:\le\:$ 60 or $\:>$ 60), sex, monthly family income in USD, self-reported smoking (smoker or non-smoker), and other chronic illnesses.

Weight was measured on a high-precision battery-powered Cadence^®^ scale, with a liquid crystal display and a maximum weight of 150 kg. The participants were weighed in light clothing, barefoot, and standing in the center of the scale. A Cescorf^®^ anthropometer was fixed to a smooth wall without a baseboard to measure the height. The movable rod was graduated in centimeters and millimeters. Body mass index was calculated as the weight in kilograms divided by the square of the height in meters.

Blood pressure was measured using an Omron^®^ Hem 742 automatic blood pressure monitoring device on the right arm, with the patient sitting in a silent office after 5 min of rest. Values for fasting glucose, total cholesterol, high-density lipoprotein cholesterol, low-density lipoprotein cholesterol, and triglycerides were collected. All data were collected during routine appointments. The time limit for collecting the laboratory test results from the medical record was < 3 months before the consultation date.

The sample size was calculated in WinPepi 11.65, considering a significance level of 5%, a power of 80%, a 0.6 difference in mean PhA values between the high- and low-risk groups according to Framingham score, sample size ratio = 1, and standard deviation (SD) of 0.6 and 0.7 for low-risk and high-risk patients, respectively, following Portugal et al. [20]. Based on these parameters, 38 participants were required, 19 each for the high-risk and low-risk groups. The sample size was increased to 160 participants, accounting for adjustments related to sex, age, and multiple analyses.

Participant characteristics are presented as mean (SD) or median (IQR) for continuous variables. To assess the association between PhA values ​​(outcome variable), Framingham score, and HEARTS score, we used generalized linear regression models, both unadjusted and adjusted for sex and age. The low-risk group was the reference for comparisons in terms of scores. We also assessed the association between PhA values and exposure variables, hypertension (yes or no), smoking (yes or no), diabetes (yes or no), metabolic syndrome (yes or no), dyslipidemia (yes or no), statin use (yes or no), and blood glucose (mg/dl) through generalized linear regression models, both unadjusted and adjusted for sex and age. The statistical analysis was performed in IBM SPSS Statistics 21.0 (I.B.M., Armonk, NY, U.S.A.). All tests were two-sided, and *P*-values < 0.05 were considered significant.

## Results

A total of 167 patients were eligible for the study, of whom three were excluded: 2 withdrew after metals were found in their bodies, and the third asked to be removed. The mean participant age was 52.2 (SD 17.9) years. Most were female 102 (62%) and declared their race white 102 (64%). The median monthly family income was USD 948.37 (Table 1).

Almost half of the patients had hypertension 76 (46.3%), approximately one-quarter had diabetes 45 (27.4%), and approximately one-third had dyslipidemia 59 (36%). Nine (5.5%) were diagnosed with renal failure, and 24 (15%) were diagnosed with metabolic syndrome. Approximately one-tenth of the participants smoked 23 (14%) (Table 1).

According to the Framingham score, 49 (31.2%) patients were at high risk of a cardiovascular (CV) event, while according to the HEARTS score, 62 (38%) were at high risk. Most patients were classified as low-risk according to Framingham and HEARTS scores 59 (37.6%) and 75 (46%), respectively (Table 1).

**Table 1:** Sociodemographic, Clinical, and Phase Angle Sample characteristics

Age	
Mean ± SD^a^	52.2 ± 18.0
Sex, *N* (%)	
Men	62 (37.8)
Women	102 (62.2)
Self-declared skin color, *N* (%)	
White	102 (64.2)
Black, Brown, Indigenous	57 (35.8)
**Family Income ($)**	
Median (Interquartile Range)	909.0 (227–1.818)
**Body Mass Index (kg/m** ^**2**^ **)**	
Mean ± SD	28.5 ± 6.31
**Hypertension**,** N (%)**	76 (46.3)
**Diabetes**,** N (%)**	45 (27.4)
**Smoking Status**,** N (%)**	
Current smokers	23 (16)
Never smokers	121 (84)
**Metabolic Syndrome**,** N (%)**	24 (15)
**Dyslipidemia**,** N (%)**	59 (36)
**Renal Failure**,** N (%)**	9 (5.5)
**Framingham General Risk Score**,** N (%)**	
Low Risk	59 (37.6)
Intermediate Risk	49 (31.2)
High Risk	49 (31.2)
**Hearts Cardiovascular Risk Calculator**,** N (%)**	
Low Risk	75 (46)
Intermediate Risk	26 (16)
High/Very High-Risk	62 (38)
**Phase Angle (kHz)**	
**Mean ± SD**	6.12 ± 1.10

^a^*SD* Standard deviation

In the univariate analysis, the Framingham low-risk group had a higher mean PhA than the intermediate-risk (ß = -0.57 [95% CI -0.95 to -0.19], *P* = 0.003) and high-risk groups (ß = -0.98 [95% CI -1.37 to -0.60], *P* < 0.001). In the multivariate analysis, there was a trend towards significance for a higher mean PhA in the low-risk group than the high-risk group (ß = -0.43 [95% CI -0.88 to 0.02], *P* = 0.06) (Table 2).

In the univariate analysis, the HEARTS low-risk group had a higher mean PhA than the high/very-high risk group (ß = -1.06 [95% CI -1.39 to -0.73], *P* < 0.001). In the multivariate analysis, the PhA values of the HEARTS low-risk group remained significantly higher than the high/very high-risk group (ß = -0.57 [95% CI -0.95 to -0.19], *P* = 0.003) (Table 2).

In the univariate analysis, participants with hypertension had lower PhA values than those without it (ß = -0.77 [95% CI -1.09 to -0.46], *P* < 0.001), as did people with diabetes compared to non-diabetics (ß = -0.88 [95% CI -1.24 to -0.53], *P* < 0.001). Participants with metabolic syndrome (ß = -0.80 [95% CI -1.26 to -0.35], *P* < 0.001), dyslipidemia (ß = -0.41 [95% CI -0.76 to -0.07], *P* = 0.02), and statin users (ß = -0.54 [95% CI -0.89 to -0.19], *P* = 0.002) also had lower PhA values than controls. There was an inverse relationship between blood glucose and PhA values (ß = -0.01 [95% CI -0.01 to 0.00], *P* = 0.003) (Table 2).

In the multivariate analysis, participants with hypertension and diabetes had lower PhA values than those without these comorbidities (ß = -0.31 [95% CI -0.62 to 0.00], *P* = 0.048 and ß = -0.49 [95% CI -0.81 to -0.16], *P* = 0.003, respectively], as did those with metabolic syndrome (ß = -0.50 [95% CI -0.90 to -0.10], *P* = 0.015). Smokers had lower mean PhA values than non-smokers (ß = -0.41 [95% CI -0.81 to -0.01], *P* = 0.040). The inverse relationship between blood glucose and PhA values remained (ß = -0.004 [95% CI -0.01 to 0.00], *P* = 0.033) (Table 2).

**Table 2:** Differences between means of phase angle values and risk scores, risk factors, statin use, and blood glucose values

	**Univariate Analysis**						
	**Phase Angle**						
	**Mean**	**Lower**	**Upper**	**Exp(ß)**	**Lower**	**Upper**	**P**
**F.R.S.**							
High	5.61	5.33	5.89	-0.99	-1.37	-0.61	**< 0.001**
Intermediate	6.03	5.75	6.31	-0.57	-0.95	-0.19	**0.003**
Low	6.60	6.34	6.86	1			
**Hearts CVD Risk**							
High/Very High	5.51	5.27	5.76	-1.06	-1.39	-0.73	** < 0.001**
Intermediate	6.25	5.88	6.63	-0.32	-0.76	0.12	0.151
Low	6.58	6.35	6.80	1			
**Hypertension**							
Yes	5.71	5.48	5.94	-0.77	-1.09	-0.46	** < 0.001**
No	6.48	6.27	6.67	1			
**Smoking**							
Yes	6.42	5.97	6.87	0.27	-0.22	0.76	0.275
No	6.144	5.948	6.34	1			
**Diabetes**							
Yes	5.48	5.18	5.78	-0.88	-1.24	-0.53	** < 0.001**
No	6.36	6.18	6.55	1			
**Metabolic Syndrome**							
Yes	5.43	5.01	5.85	-0.80	-1.26	-0.35	**0.001**
No	6.23	6.1	6.41	1			
**Dyslipidemia**							
Yes	5.86	5.58	6.13	-0.41	-0.76	-0.07	**0.02**
No	6.27	6.06	6.48	1			
**Statins**							
Yes	5.77	5.48	6.04	-0.54	-0.89	-0.19	**0.002**
No	6.30	6.10	6.50	1			
**Blood Glucose (mg/dL)**	107.8	69	369	-0.006	-0.01	-0.002	**0.003**
	**Multivariate Analysis**^**a**^						
	**Phase Angle**						
	**Mean**	**Lower**	**Upper**	**Exp(ß)**	**Lower**	**Upper**	**P**
**F.R.S.**							
High	5.85	5.58	6.11	-0.43	-0.88	0.02	0.06
Intermediate	6.13	5.87	6.39	-0.14	-0.53	0.24	0.46
Low	6.27	5.97	6.58	1			
**Hearts CVD Risk**							
High/Very High	5.73	5.50	5.96	-0.57	-0.95	-0.19	**0.003**
Intermediate	6.40	6.06	6.74	0.09	-0.35	0.54	0.682
Low	6.30	6.04	6.57	1			
**Hypertension**							
Yes	5.91	5.70	6.12	-0.31	-0.62	-0.00	**0.048**
No	6.22	6.01	6.44	1			
**Smoking**							
Yes	6.06	5.89	6.24	-0.41	-0.81	-0.01	**0.04**
No	6.47	6.08	6.86	1			
**Diabetes**							
Yes	5.72	5.46	5.99	-0.49	-0.81	-0.16	**0.003**
No	6.21	6.03	6.39	1			
**Metabolic Syndrome**							
Yes	5.64	5.28	6.00	-0.50	-0.90	-0.10	**0.015**
No	6.14	5.97	6.30	1			
**Dyslipidemia**							
Yes	6.05	5.81	6.29	-0.03	-0.33	0.28	0.87
No	6.07	5.88	6.27	1			
**Statins**							
Yes	6.01	5.76	6.25	-0.09	-0.42	0.23	0.57
No	6.10	5.90	6.29	1			
**Blood Glucose (mg/dL)**	107.8	69	369	-0.004	-0.007	0	**0.033**

*Abbreviations:* *F.R.S.* Framingham Risk Score, *Hearts CVD Risk* Hearts Cardiovascular Risk Calculator

^a^Adjusted for sex and age

## Discussion

The present study evaluated the association between PhA and cardiovascular risk. Lower PhA values were associated with higher cardiovascular risk according to the HEARTS score, and there was also a trend towards significance for lower PhA values to be associated with high risk according to the Framingham score.

Risk scores involve factors such as age, hypertension, diabetes, and smoking associated with chronic inflammatory processes [27]. It has already been shown that chronic inflammation is associated with lower PhA values [28], and it is also recognized as a contributing factor to numerous chronic diseases, including CVD [20, 28]. The interplay of inflammation, cell death, and fibrosis plays a critical role in the progression of CVD, disrupting both intra- and extracellular water balance and resulting in membrane damage [28–31]. These mechanisms ultimately alter the electrical conductivity of tissues, thereby impacting PhA. Furthermore, evidence suggests that oxidative stress and immune pathways are a primary mechanism in many CVDs, leading to cellular damage and death, which further contributes to the association with reduced PhA values [17].

Our results are similar to those of previous studies, which showed an inverse association between the Framingham risk score and PhA, with the results adjusted for sex [19, 20]. However, to the best of our knowledge, regarding the HEARTS score, which has been validated for Latin America, ours is the first study to assess its association with PhA [6].

Our study also found an association between sex- and age-adjusted PhA and independent risk factors, like other studies evaluating its association with hyperglycemia [32, 33], smoking [34], diabetes [32, 33, 35, 36], and metabolic syndrome [37], in addition to dyslipidemia and hypertension. The mechanisms that justify this association are similar to those for total scores since these factors are associated with the inflammatory process, which causes oxidative stress and cell damage, which can consequently reduce PhA [38, 39].

One limitation of this study is that for participants whose age was above or below the age range for the risk calculator, the calculation was performed using the upper or lower age limit. However, we expect that this can be performed routinely [40], bringing our study’s results closer to clinical practice. The present study aimed to relate different tools for assessing cardiovascular risk simultaneously with convenience samples in the clinical environment. The association between these tools underscores the importance of conducting cohort studies across diverse populations and health scenarios to evaluate PhA as a cardiovascular prognostic assessment tool. The lack of consideration of other potential confounding factors may be mitigated by the fact that the associated measurements were conducted on the same subjects during the same period.

## Conclusion

The present study shows that individuals at high cardiovascular risk have lower mean PhA values than those at low risk. To enhance the generalizability of these findings, it is crucial to conduct future cohort studies in more controlled settings, with patients in different measurement evaluation positions encompassing a diverse range of populations and health scenarios.

## Data Availability

The datasets used and/or analysed during the current study are available from the corresponding author on reasonable request.

## Abbreviations

CV: Cardiovascular
CVD: Cardiovascular Disease
F.R.S.: Framingham Risk Score
Hearts CVD Risk: Hearts Cardiovascular Risk Calculator
PhA: Phase Angle
